# Oxidative Degradation of Chitosan to the Low Molecular Water-Soluble Chitosan over Peroxotungstate as Chemical Scissors

**DOI:** 10.1371/journal.pone.0100743

**Published:** 2014-06-27

**Authors:** Zhanwei Ma, Wenyan Wang, Ying Wu, Yiming He, Tinghua Wu

**Affiliations:** 1 Key Laboratory of the Ministry of Education for Advanced Catalysis Materials, Institute of Physical Chemistry, Zhejiang Normal University, Jinhua, People's Republic of China; 2 Department of Material Physics, Zhejiang Normal University, Jinhua, People's Republic of China; Queen's University Belfast, United Kingdom

## Abstract

Low molecular water-soluble chitosan was prepared by the depolymerization of chitosan in the presence of a series of catalysts with active W(O_2_) sites. Both the peroxo species [W_2_O_3_(O_2_)_4_]^2-^ and {PO_4_[WO(O_2_)_2_]_4_}^3-^ showed high efficiency in the degradation of chitosan, indicating that the degradation mechanism did not follow the radical mechanism. That means •OH is not the active species, which has been proven by the fluorescence spectra. H_2_O_2_ acted as an oxidant to regenerate the active W(O_2_) sites in the depolymerization of chitosan. The developed catalyst (TBA)_3_{PO_4_[WO(O_2_)_2_]_4_} is recoverable.

## Introduction

**Figure 1:**
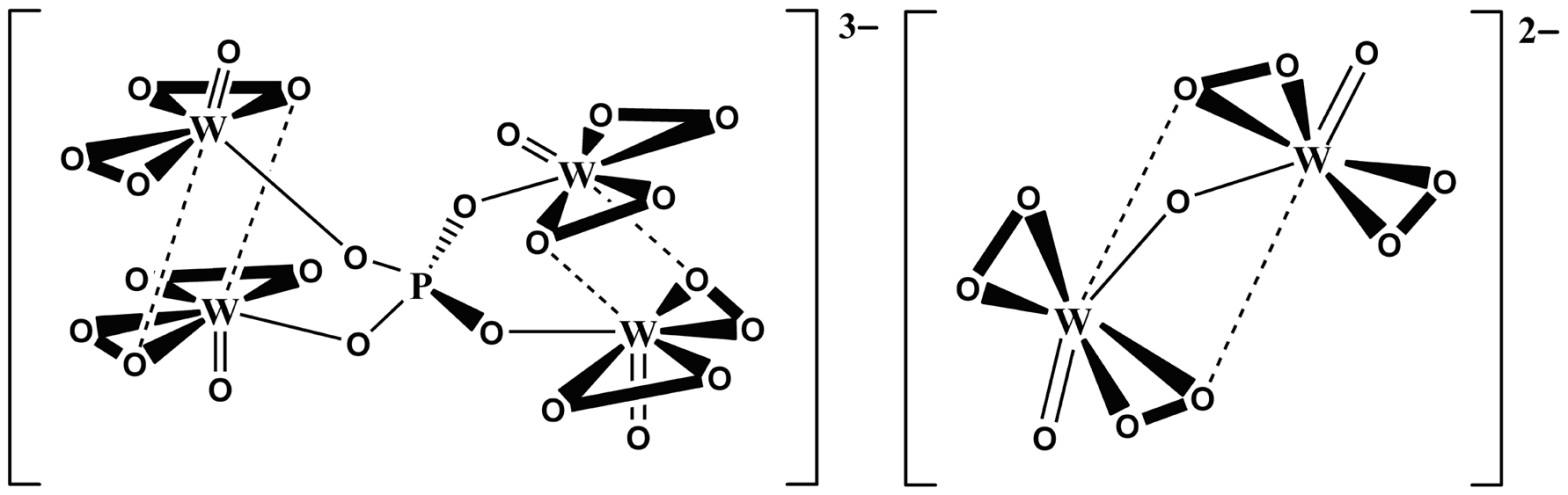
The chemical structure of peroxo species.

Chitosan is a biopolymer obtained by partial deacetylation of chitin, which is the second most abundant polysaccharide in nature after cellulose[1], [2]. However, chitosan has a high molecular weight and low solubility in most solvents, which limits its applications in agriculture[3], [4], food and beverages[5], waste water treatment[6]-[8], pharmaceuticals[9], [10] and biomaterials[11]. By the degradation process chitosan can be converted into the low-molecular-weight chitosan which exhibits good water solubility. To date, acid hydrolysis, basic hydrolysis and oxidation-based methods are commonly used for the preparation of the low-molecular water-soluble chitosan (LMWSC). Catalytic oxidation with hydrogen peroxide is particularly attractive from the economical and environmental benefits. However, oxidative degradation of chitosan using H_2_O_2_ without additives is inefficient. Thus, the combined methods for degradation of chitosan by using H_2_O_2_ and chemical or physical techniques, such as the catalysis of Fe^2+^, Cu^2+^, heterpoly compounds, ultraviolet, microwave irradiation and gamma radiation, have been studied[12]–[15]. Recently, Huang et al. reported that a Keggin heterpoly acid, H_3_PW_12_O_40_, can efficiently catalyze oxidative degradation of chitosan with H_2_O_2_ to the LMWSC[16]–[18]. The authors presumed that 1) the peroxo species {PO_4_[WO(O_2_)_2_]_4_}^3−^ formed by phosphotungstic acid in the presence of H_2_O_2_ is the real catalyst. 2) the peroxo species {PO_4_[WO(O_2_)_2_]_4_}^3−^ accelerates the formation of free radicals by the disassociation of hydrogen peroxide. 3) the free radicals can attack the β-(1–4) glucosidic bond of chitosan to obtain the LMWSC. To the best of our knowledge, however, little experimental data is available to support this conjecture. Besides, some researchers[19], [20] have reported that heteropolyacids with the Keggin structure (H_3_PW_12_O_40_) are degraded in the presence of H_2_O_2_ to form two peroxo species {PO_4_[WO(O_2_)_2_]_4_}^3−^ and [W_2_O_3_(O_2_)_4_]^2−^ (**Figure 1**). These results motivated us to reconsider why the real catalyst is not [W_2_O_3_(O_2_)_4_]^2−^ but the peroxo species {PO_4_[WO(O_2_)_2_]_4_}^3−^, also it is not clear why the peroxo species [W_2_O_3_(O_2_)_4_]^2−^ formed from H_3_PW_12_O_40_ showed no catalytic activity.

In this report, the isolated peroxo species (TBA)_3_{PO_4_[WO(O_2_)_2_]_4_} and K_2_[W_2_O_3_(O_2_)_4_] were directly employed as the catalyst to degrade chitosan. We demonstrated that not only the peroxo species {PO_4_[WO(O_2_)_2_]_4_}^3−^, but also [W_2_O_3_(O_2_)_4_]^2−^ are highly efficient catalysts. The peroxo species itself can efficiently degrade chitosan without hydrogen peroxide. Moreover, the (TBA)_3_{PO_4_[WO(O_2_)_2_]_4_} catalyst can be recycled.

## Experiment Section

### Materials

H_3_PW_12_O_40_, Na_3_PW_12_O_40_, K_2_WO_4_, (NH_4_)_2_WO_4_, Na_2_WO_4_, EDTA, KSCN, SnCl_2_ and TiCl_3_ (AR) were purchased from Aladdin Industrial Corporation. H_2_O_2_ (Hydrogen peroxide, 30%) and Chitosan (DD≥90%) were purchased from Sinopharm Chemical Reagent Co., Ltd. The water used was distilled.

### Synthesis of (TBA)_3_{PO_4_[WO(O_2_)_2_]_4_}

The (TBA)_3_{PO_4_[WO(O_2_)_2_]_4_} was prepared according to the literature[19], [21]. Hydrogen peroxide (30%) (10 mL, 100 mmol) was added to a solution of H_3_PW_12_O_4_ (1.65 g in 1 mL water). After 30 min, an aqueous solution of tetrabutylammonium chloride (1.6 mmol in 3 mL) was slowly added. The resulting white precipitate was filtered out, washed several times with water, and then air dried. IR(KBr): *ν*(PO_4_):1084, 1053 cm^−1^, *ν*(W = O): 964 cm^−1^, *ν*(O–O): 845 cm^−1^, *ν*_asym_[W(O)_2_]: 593 cm^−1^, *ν*_sym_ [W(O)_2_]: 523 cm^−1^. Raman (in CH_3_CN): *ν*(PO_4_):1050, 1024 cm^−1^, *ν*(W = O): 957 cm^−1^, *ν*(O–O): 848 cm^−1^, *ν*_asym_[W(O)_2_]: 591 cm^−1^, *ν*_sym_ [W(O)_2_]: 520 cm^−1^.

### Synthesis of K_2_[W_2_O_3_(O_2_)_4_]

K_2_[W_2_O_3_(O_2_)_4_] was prepared according to the literature[19]–[21]. A solution of K_2_WO_4_ (5.0 g in 10 mL water) was placed in an ice-water bath. 30% H_2_O_2_ (10 mL) was slowly added, and the solution turned light yellow. Dilute hydrochloric acid was added into the solution until the color just disappeared at pH = 2∼3. Then the ethanol was added. The resulting white precipitate was filtered out, washed several times with ethanol and dried in air. IR (KBr): *ν*(W = O): 966 cm^−1^, *ν*(O–O): 854 cm^−1^, *ν*_asym_[W(O)_2_]: 550 cm^−1^, *ν*_sym_ [W(O)_2_]: 615 cm^−1^, *ν*_asym_[W_2_O]: 764 cm^−1^. Raman (solid): *ν*(W = O): 958 cm^−1^, *ν*(O–O): 861 cm^−1^, *ν*_asym_[W(O)_2_]: 553 cm^−1^, *ν*_sym_ [W(O)_2_]: 537 cm^−1^, *ν*_asym_[W_2_O]: 763 cm^−1^, *ν*_sym_[W_2_O]: 450 cm^−1^.

### The typical procedure of the degradation of chitosan

In a typical reaction, a mixture of 0.200 g of chitosan, 4.2 µmol tungsten (W) in the catalyst, 5 mL of the distilled water, 1 mL of 30% H_2_O_2_, were stirred in the 50 mL round-bottomed flask for 20 min at different temperature. After the reaction, NaOH was added to the filtrate until pH = 7. The products were extracted with ethanol. The obtained white precipitate was filtered out, dried in vacuum and then analyzed.

### Determination of tungsten

The filtrate was evaporated and calcinated at 400°C. Then the samples were digested by microwave with the digestion solvent of 40 mL NaOH solution (0.025 g/mL). After being digested for 30 min by medium-high level microwaves, the sample was cooled to room temperature, and 1 g EDTA and 30 mL deionized water were added into the beaker. Then the solution was transferred into 100 mL volumetric flask (**1**).

2 mL KSCN (0.5 g/mL), 4.5 mL SnCl_2_ (0.008 g/L) and 1.5 mL TiCl_3_ (0.15 g/mL) were added into 10 mL (**1)** solution, transferred 25 mL volumetric flask. Then the solution was measured on the Spectrophotometer 721 at λ = 406 nm.

### Analytical method

The degradation ratio of chitosan was defined as follows:
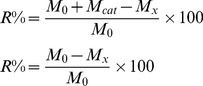
(1)where R refers to the degradation ratio of chitosan, M_0_ refers to the quantity of the original chitosan, M_cat_ refers to the quantity of the catalyst, M_x_ refers to the quantity of the collected solid after degradation under different conditions.

The Ubbe-lodhe viscosimeter was used to determine the intrinsic viscosities at 303 K. Chitosan was dissolved in 0.1mol/L CH_3_COONa–0.2 mol/L CH_3_COOH solution. The viscosity average molecular weight (M_v_) was calculated according to the following equation[16]–[18].
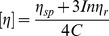
(2)

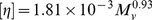
(3)


Here,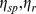 refer to the incremental viscosity and the relative viscosity respectively, C is the concentration of the LMWSC (g/mL). M_v_ is the viscosity average molecular weight.

The FT-IR spectra of samples were recorded on a NEXUS 670 FT-IR spectrometer with KBr pellets prepared by manual grinding. Raman spectra of samples were recorded on Renishaw RM1000 (λ = 514.5 nm). Fluorescence spectra were acquired with an FLSP920 spectrofluorometer (Edinburgh Instruments Ltd, UK) at 20±1°C, equipped with a temperature-controlled circulator (Julabo, Germany). X-ray diffraction (XRD) patterns of samples were carried out on a PW 3040/60 X-ray diffractometer (Philips, Netherlands) with Cu Kα target at 40 kV and 40 mA. The samples were scanned from 5°to 70°of 2θ. The UV-vis absorption spectra of the samples were measured in the range of 100–800 nm on a UV-vis spectrometer (Nilcolet Evolution 500, Thermo). The metal content of the product solution was measured on the Spectrophotometer 721 (Shanghai Analysis Instrument Company).

## Results and Discussion

### The characterization of chitosan

**Figure 2:**
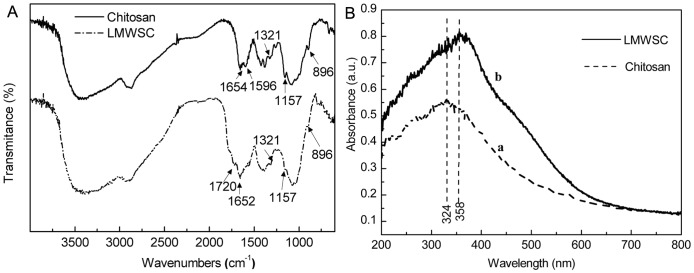
The characterization of chitosan and the LMWSC, FTIR spectra (A) and DRS patterns (B).

The chitosan and the LMWSC were characterized by FTIR in **Figure 2A**. The main bands of chitosan were as follows. The band at around 3422 cm^−1^ could be ascribed to the stretching vibration of O-H and N-H. The absorption peak at 1599 cm^-1^ corresponds to the binding vibration of the amido groups. An apparent carboxyl (-C = O) band at 1654 cm^−1^ is attributed to the residual acetyl. The band in the range 1157 cm^−1^ to 896 cm^−1^ belongs to the special absorb peaks of β-1,4 glycoside bond in chitosan[22]. The similar characteristics in the FTIR spectrum of the LMWSC can also be observed. Besides, a new absorption peak at 1720 cm^−1^ is observed in the spectrum of the LMWSC, which is assigned to the absorption of the carboxylic group (–COOH). It indicates the existence of the carboxylic group in the LMWSC.

To further identify the carboxylic group of the LMWSC, DRS analysis are given in **Figure 2B**. There is an absorption band at 324 nm in the original chitosan, which is caused by the n→π* transition of residual acetyl (DD>90%). Compared with that of chitosan, the DRS spectrum of the LMWSC has a new absorption band at 357 nm caused by the n→π* transition of a carboxylic group in the LMWSC. Consequently, the DRS patterns reflected that the carboxylic group was formed, which coincided with the analysis of the FTIR spectra.

**Figure 3:**
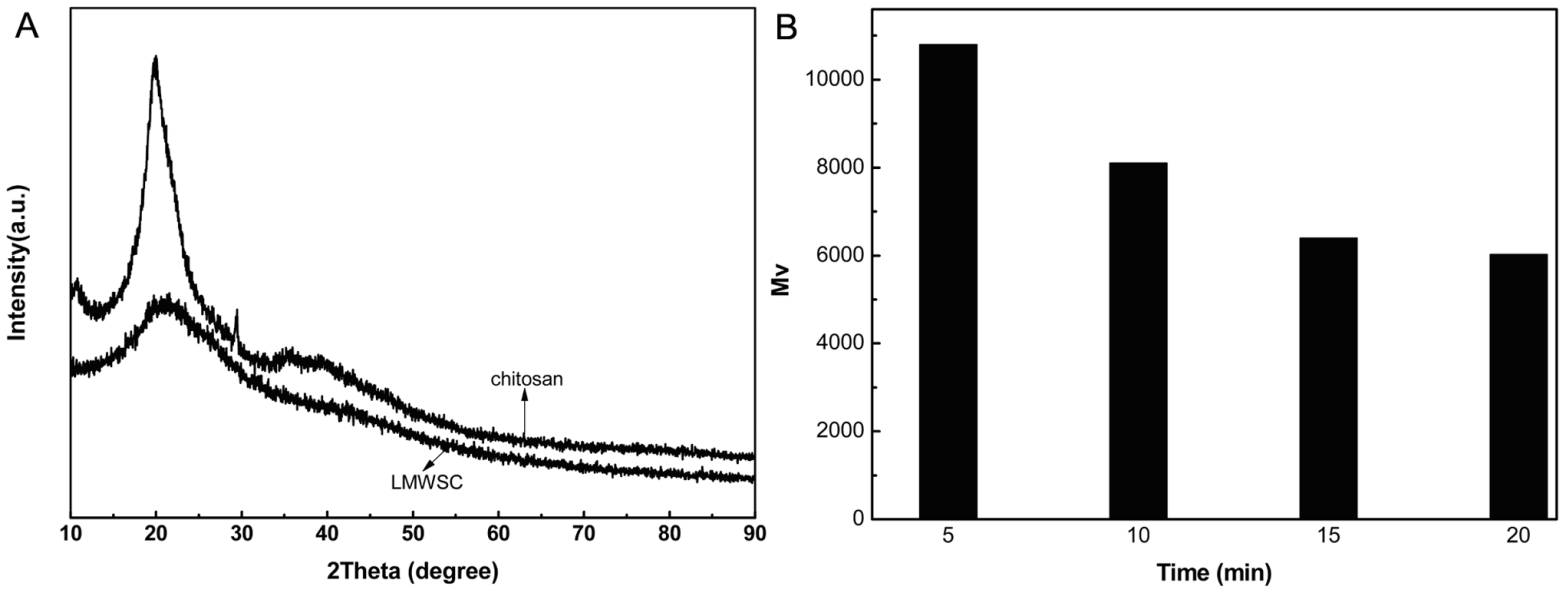
The characters of chitosan and the LMWSC, XRD patterns (A), and the effect of the reaction time on the Mv of LMWSC (B).

The X-ray powder patterns of chitosan and the LMWSC are shown in **Figure 3A**. The pattern of chitosan shows two characteristic peaks at 2θ  =  10.4° and 20.0° which correspond to (1 0 0) and (0 2 0) reflections of the L-2 polymorph of chitosan[23]. For the LMWSC, the peak at 2θ  =  10.4° disappeared, and the strength of the peak at 2θ  =  20.0° decreased. It indicated the crystalline structure of the LMWSC was destroyed and the crystallinity decreased. LMWSC became more amorphous than chitosan[24]. It manifests that the degradation of chitosan occurred preferentially from the amorphous region to the water-soluble molecular, which dissolved in the water. With the further degradation, the crystalline structure was destroyed thoroughly. **Figure 3B** shows the viscocity average molecular weight (Mv) of the LMWSC versus time on the (TBA)_3_{PO_4_[WO(O_2_)_2_]_4_} catalyst. After 20min the Mv of theLMWSC can reach 5892.

### The catalytic activities of the catalysts

**Figure 4:**
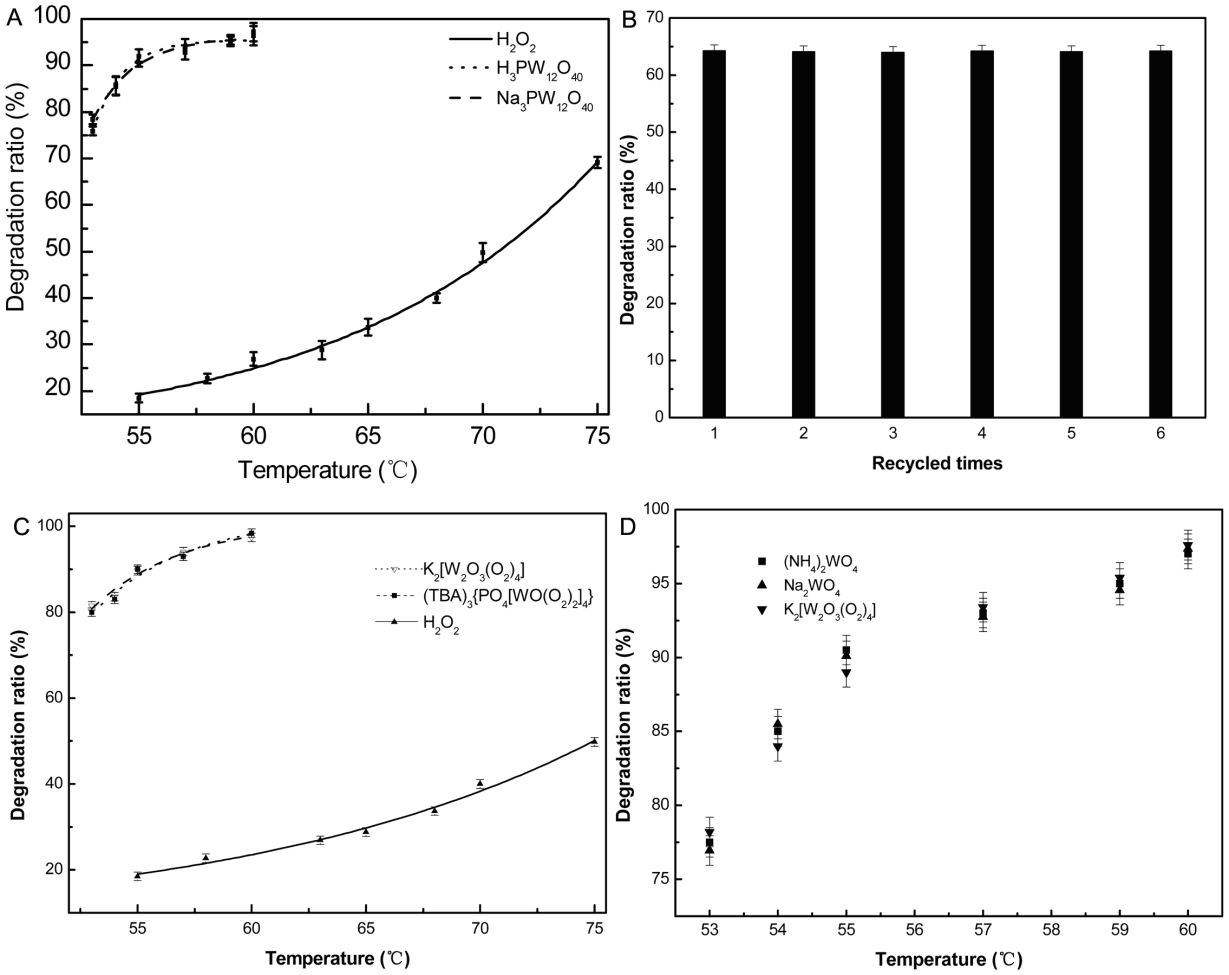
The oxidative degradation of chitosan, (A) H_3_PW_12_O_40_ (0.35 µmol), Na_3_PW_12_O_40_ (0.35 µmol) and H_2_O_2_ without any additives. (C) (TBA)_3_{PO_4_[WO(O_2_)_2_]_4_} (1.05 µmol), K_2_[W_2_O_3_(O_2_)_4_] (2.1 µmol) and H_2_O_2_ without any additives. (D) Na_2_WO_4_ (4.2 µmol), (NH_4_)_2_WO_4_ (4. 2 µmol) and K_2_[W_2_O_3_(O_2_)_4_] (2.1 µmol). Under the same reaction conditions: H_2_O_2_ (1 mL), H_2_O(5 mL), 20 min. (B) Recycling of the filtrate at 65°C, 1 mL H_2_O_2_.

We first reinvestigated the oxidative degradation of chitosan with H_3_PW_12_O_40_ and H_2_O_2_. Compared with H_2_O_2_, the degradation efficiency increased significantly (**Figure 4A**). This could be expected considering that both the acid hydrolysis of chitosan and the oxidative dagredation of chitosan might occur in the presence of H_3_PW_12_O_40_. Thus, we switched to the salt Na_3_PW_12_O_40_. Interestingly, the degradation ratio of chitosan is not significantly changed when three Na^+^ substituted the position of protons in H_3_PW_12_O_40_, which allowed us to exclude the contribution of Brønsted acid catalysis for the reaction. In an attempt to interpret the results, we added an aqueous solution of tetrabutylammonium chloride to the solution of H_3_PW_12_O_40_ and 30% H_2_O_2_. The obtained white precipitate (TBA)_3_{PO_4_[WO(O_2_)_2_]_4_} was filtered out. It is worthwhile to note that the filtrate is still active for the degradation of chitosan although the catalytic activity is low. And most interestingly, the filtrate can be reused six times without loss of reactivity (**Figure 4B**). The results can be explained if (TBA)_3_{PO_4_[WO(O_2_)_2_]_4_} is not completely filtered out or the peroxo species [W_2_O_3_(O_2_)_4_]^2−^ dissolved in the filtrate is also an active species. Therefore, we turned our attention to the two peroxo species {PO_4_[WO(O_2_)_2_]_4_}^3−^ and [W_2_O_3_(O_2_)_4_]^2−^, which are always formed in the presence of H_2_O_2_.

### The catalytic activity of the peroxo species

Next, we examined the oxidative degradation of chitosan by (TBA)_3_{PO_4_[WO(O_2_)_2_]_4_} and K_2_[W_2_O_3_(O_2_)_4_] with H_2_O_2_, seperately. The results are shown in **Figure 4C**. We can see that the degradation ratio of chitosan is still high for the peroxo species, while only a very small amount of chitosan is degraded in the absence of peroxo species. Most interestingly, no significant change in the degradation ratio of chitosan was observed when (TBA)_3_{PO_4_[WO(O_2_)_2_]_4_} (the content of W atom: 4.2 µmol) and K_2_[W_2_O_3_(O_2_)_4_] (the content of W atom: 4.2 µmol) were used. The catalyst (TBA)_3_{PO_4_[WO(O_2_)_2_]_4_} is almost insoluble and K_2_[W_2_O_3_(O_2_)_4_] is highly soluble in water. By further investigation we found 1.05 µmol (TBA)_3_{PO_4_[WO(O_2_)_2_]_4_} was not soluble in 5 mL water at room temperature. However, when heated to 50°C, the catalyst (TBA)_3_{PO_4_[WO(O_2_)_2_]_4_} was soluble in water. When cooled to room temperature, it precipitated as a solid. Increasing the amount of the catalyst (10 µmol) in 5 mL water, part of the catalyst was still not soluble when heated at 50°C. Thus, we can conclude that the catalyst ascribed to the ionic compound has a certain solubility in the water when the water was heated, so that (TBA)_3_{PO_4_[WO(O_2_)_2_]_4_} and K_2_[W_2_O_3_(O_2_)_4_] with the same W content gaved almost the same activities under the present reaction condition. The results also motivated us to revisit the structure of the two peroxo species (**Figure 1**). Both of the peroxo species possess the same unit of W(O_2_). It indicates that the unit of W(O_2_) might be the active sites for oxidative degradation of chitosan. In order to prove the above suggestion, Na_2_WO_4_ or (NH_4_)_2_WO_4_ with H_2_O_2_ which could also form the active W(O_2_) sites[21], [25] were used as the catalysts to degrade chitosan. As shown in **Figure 4D**, the two samples exhibit the degradation ratio of chitosan as high as that of K_2_[W_2_O_3_(O_2_)_4_], which is consistent with our suggestion of the W(O_2_) active sites.

**Figure 5:**
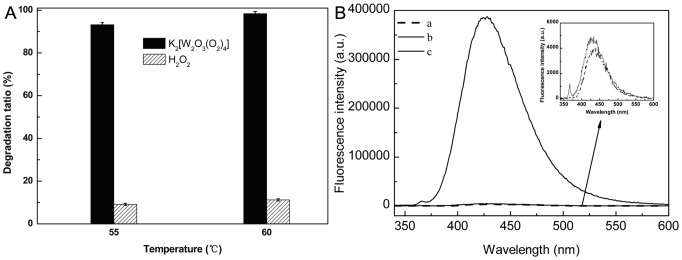
The comparison of different peroxo species, the peroxo species K_2_ [W_2_O_3_(O_2_)_4_] (2.5 mmol) and H_2_O_2_ (10 mmol), respectively, under the same reaction conditions: 5 mL H_2_O, 20 min (A), and the Fluorescence spectra changes (excitation at 315nm) with different addtives (B), a. terephthalic acid (5 ×10^−5^ M), Na_2_WO_4_ (4.2 µmol), H_2_O_2_ solution (100 µL) b. terephthalic acid (5 ×10^−5^ M) and H_2_O_2_ solution (100 µL). c. terephthalic acid (5 ×10^−5^ M), K_2_[W_2_O_3_(O_2_)_4_] (2.1 µmol), H_2_O_2_ solution (100 µL), under the same reaction conditions: 55°C, 2 min.

Except where noted, we are interested to know whether the K_2_[W_2_O_3_(O_2_)_4_] containing the active W(O_2_) sites can behaved as the efficient oxidant without H_2_O_2_ in the preparation of LMWSC. Therefore, some comparative experiments were carried out and the results are summarized in **Figure 5A**. It is clear that the degradation ratio of chitosan was high with K_2_[W_2_O_3_(O_2_)_4_] in the absence of H_2_O_2_, while that was low with only H_2_O_2_. Moreover, the results further supported the hypothesis that the structure of W(O_2_) is the real active site in the degradation of chitosan because both of them contain an equal quantity of the peroxo group(10 mmol). To the best of our knowledge, this is the first example of highly efficient utilization of the peroxo group to obtain the LMWSC. The peroxo species K_2_[W_2_O_3_(O_2_)_4_] itself was more efficient for the degradation of chitosan than hydrogen peroxide. This intriguing finding shows that we need to know more about the oxidative degradation of chitosan by K_2_[W_2_O_3_(O_2_)_4_] with H_2_O_2_.

### The mechanism of the degradation of chitoan

**Figure 6:**
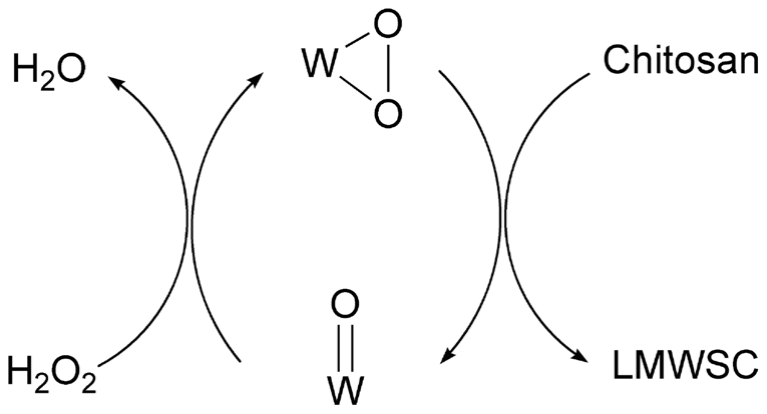
The mechanism of degradation of chitosan.

It has been well documented in previous work that the unit of W(O_2_) is the active sites for oxidative different substrate such as epoxidation of olefins[26], [27], desulfurization of fuels[28], and oxidation of alcohols[29]. Our work also lead to the conclusion that W(O_2_) structures are the active sites. There are efficient for the degradation of chitosan, even in the absence of H_2_O_2_. However, in the previous work,[13], [15] decomposition of H_2_O_2_ to free radicals is considered as a key factor in the degradation of chitosan. To further investigate the role of H_2_O_2_ in the oxidative degradation of chitosan by K_2_[W_2_O_3_(O_2_)_4_] with H_2_O_2_, the decomposition of H_2_O_2_ was detected according to the literature[30], [31]. The results were summarized in **Figure 5B**. The fluorescence signal at 426 nm can be assigned to 2-hydroxyterephthalic acid, which results from the capture of ·OH by terephthalic acid. Compared with the fluorescence of the system with only H_2_O_2_, that of the system with an extra Na_2_WO_4_ or K_2_[W_2_O_3_(O_2_)_4_] was much weaker under the same conditions(**Figure 5B** **a,c**), which indicated a low content of ·OH was in the system and the peroxo species exhibited its inherent poor activity for the decomposition of hydrogen peroxide[32]–[35]. Combining the above facts, H_2_O_2_ was suggested to act as an oxidant to re-form the structure of W(O_2_), on which chitosan was degraded. Hence, a plausible reaction mechanism might contain the active W(O_2_) sites cutting the β-1,4 glycoside bond in chitosan to obtain the LMWSC and the regeneration of the active sites (**Figure 6**).

### Recycling of the catalyst (TBA)_3_{PO_4_[WO(O_2_)_2_]_4_}

**Figure 7:**
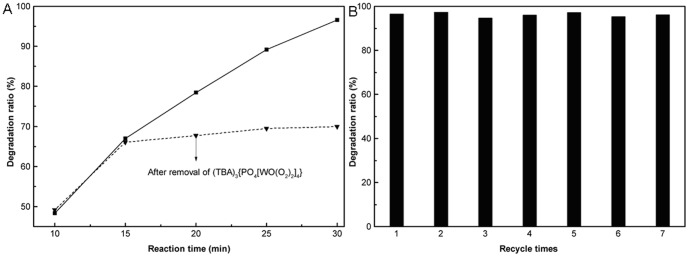
The catalytic activity of the filtrate and the catalyst. The filtrate (A) and the catalyst (B), 0.0125 mmol (TBA)_3_{PO_4_[WO(O_2_)_2_]_4_}, 1 mL H_2_O_2_, 65°C.

**Figure 8:**
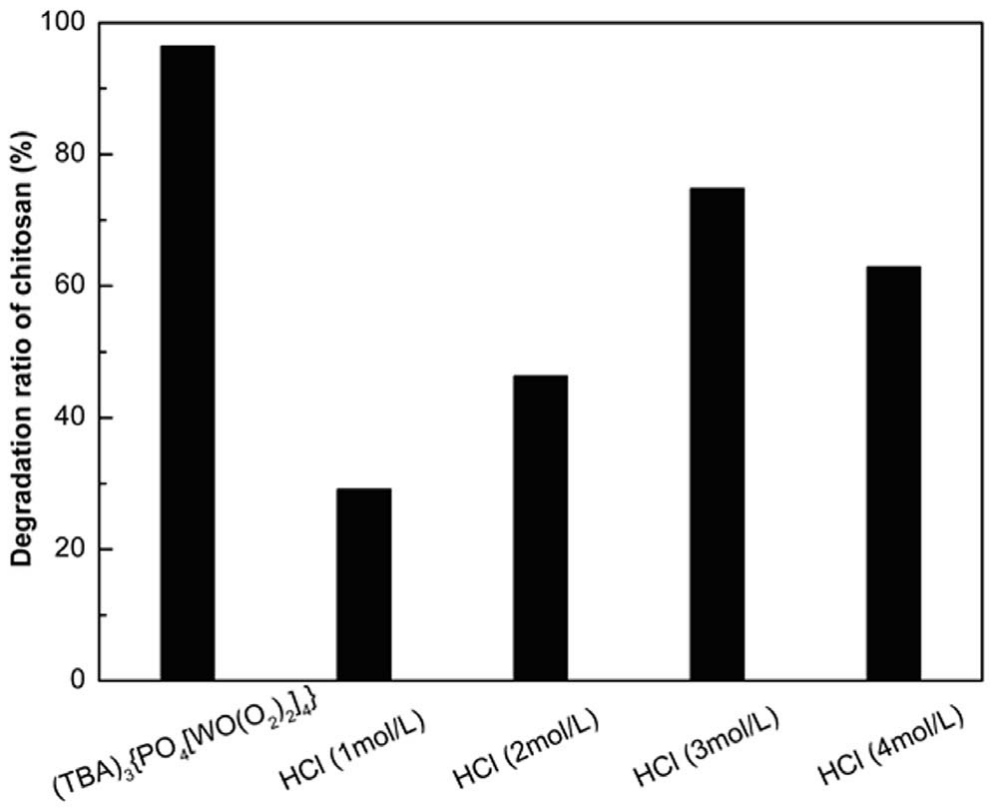
Degradation ratio of chitosan by different methods.

The catalyst K_2_[W_2_O_3_(O_2_)_4_] can be dissolved in water, which is not beneficial for the recycle of the catalyst. Hence, we turned our attention to the water insoluble catalyst (TBA)_3_{PO_4_[WO(O_2_)_2_]_4_}. When the reaction had proceeded for 15 min, the vessel was cooled to room temperature. Then the catalyst was filtered and the filtrate was used for the next run. The results were shown in **Figure 7A** **.** After removal of the catalyst, the degradation ratio of chitosan was obviously decreased, and only 0.6 µg/mL W was determined in the product solution. The degradation ratio of chitosan was slightly increased after 15 min probably due to the minute amount of W remained in the filtrate. Then, the recycling experiment of the catalyst was investigated. The results in **Figure 7B** show that the degradation ratio could still reach a high level of 96.2% after recycling 7 times. For comparison, the acid hydrolysis method was also used in the degradation of chitosan and hydrochloric acid of different concentration acid was employed (**Figure 8**). With the increase in the concentration of HCl, the degradation ratio of chitosan increased. Although the degradation ratio could reach 74.8% in 3mol/L HCl, the degradation ratio decreased to 62.9% in 4mol/L HCl. It could be ascribed that the low concentration of hydrochloric acid favors the chitosan dissolving in solution, while high concentration of hydrochloric acid restrains the chitosan dissolution[36]. What's more, large amounts of inorganic acid are not friendly for the environment. It's clear that the novel catalyst developed herein is promising for application in preparation of LMWSC.

## Conclusions

In conclusion, this study showed that the peroxo species K_2_[W_2_O_3_(O_2_)_4_] itself was more efficient for the degradation of chitosan than hydrogen peroxide. Both the peroxo species [W_2_O_3_(O_2_)_4_]^2−^ and {PO_4_[WO(O_2_)_2_]_4_} are effective catalysts for the oxidative degradation of chitosan to obtain LMWSC in water with high efficiency of hydrogen peroxide utilization. The structure of W(O_2_) was considered to be the active site. In addition, the new catalyst (TBA)_3_{PO_4_[WO(O_2_)_2_]_4_} was efficient for the degradatiion of chitosan, and the recovered catalyst could achieve a high degradation ratio of 96.2% after 7 times.
